# Initial appearance and temporal distribution of pedicle screw loosening after single-level lumbar interbody fusion

**DOI:** 10.1038/s41598-026-56800-2

**Published:** 2026-06-09

**Authors:** Munenari Ikezawa, Satoru Tanioka, Takahiro Miyazaki, Shota Ito, Atsushi Yamamoto, Hirofumi Nishikawa, Masashi Fujimoto, Fujimaro Ishida, Yusuke Kamei, Koichiro Yoshida, Motoi Shoda, Hidenori Suzuki, Masaki Mizuno

**Affiliations:** 1 Department of Neurosurgery, Mie Chuo Medical Center, 2158-5 Myojin-cho, Hisai, Tsu, Mie 514-1101 Japan; 2 Charité Lab for Artificial Intelligence in Medicine, Charité Universitätsmedizin Berlin, Charitéplatz 1, 101117 Berlin, Germany; 3 Department of Neurosurgery, Mie University Graduate School of Medicine, 2-174 Edobashi, Tsu, Mie 514-8507 Japan; 4 Department of Neurosurgery, Suzuka Kaisei Hospital, 112-1 Ko-cho, Suzuka, Mie 513- 8505 Japan; 5 Department of Neurosurgery, Kuwana City Medical Center, 3-11 Kotobuki-cho, Kuwana, Mie 511-0061 Japan; 6 Department of Neurosurgery, Japanese Red Cross Ise Hospital, 1-471-2 Funae, Ise, Mie 516-8512 Japan; 7 Department of Neurosurgery, Mie Prefectural General Medical Center, 5450-132 OazaHinaga, Yokkaichi, Mie 510-8561 Japan; 8 Department of Neurosurgery, Spine Center, Yachiyo Hospital, 2-2-7 Symiyoshi-cho, Anjo, Aichi 446-8510 Japan; 9 Department of Minimum-Invasive Neurospinal Surgery, Mie University Graduate School of Medicine, 2-174 Edobashi, Tsu, Mie 514-8507 Japan

**Keywords:** Lumbar degenerative disease, Pedicle screw loosening, Lumbar interbody fusion, Temporal distribution, Computed tomography, Anatomy, Diseases, Health care, Medical research

## Abstract

Pedicle screw (PS) loosening is a recognized complication after lumbar interbody fusion, but the timing of its initial appearance and its temporal distribution remain insufficiently defined. This retrospective study evaluated 212 patients who underwent single-level lumbar interbody fusion with PS fixation to clarify when PS loosening first appears, how it changes over time, and which clinical factors are associated with its development. The median follow-up duration was 502 days [364.5–731.75]. PS loosening was identified on postoperative computed tomography in 18.9% of patients. Approximately half of the cases were observed within 90 days, and nearly two-thirds within 180 days. Most cases occurred during the early postoperative period, but a smaller number continued to emerge up to one year. Occurrences beyond one year were rare. Older age and lower lumbar levels, particularly L4/5 and L5/S1, demonstrated significant associations with PS loosening. These findings provide a clearer understanding of the temporal characteristics of PS loosening and emphasize the importance of postoperative CT for detecting early changes. They also highlight the need for appropriate follow-up strategies to identify delayed cases that may otherwise be overlooked.

## Introduction

Lumbar interbody fusion with pedicle screws (PSs) has become a widely accepted standard procedure in spinal surgery for the treatment of lumbar degenerative disease. PSs provide rigid fixation to promote spinal fusion and enhance stability^1,2^. Despite the high success rate of this procedure, various complications have been reported, including nerve root injury, dural tears, infection, and implant-related failures, including PS loosening. This condition is frequently observed on plain radiographs or computed tomography (CT) scans and has been associated with postoperative bone fusion failure, potentially leading to significant clinical issues^2–6^.

PS loosening is not only a clinical phenomenon but also an indicator of inadequate fixation. Tokuhashi et al. reported that radiolucent zones were observed in 41.1% of cases six months postoperatively, with two-thirds of them resolving by the final follow-up at three or more years^7^. In another study, Wu et al. found that in cases where dynamic stabilization was performed for lumbar spondylosis, a radiolucent zone was detected in 19.8% of patients over an observation period of approximately three years, with 88% of these cases developing such zones within 6.6 months postoperatively^4^. However, data remain limited on when screw loosening occurs and how it changes over time, and these aspects of screw loosening are poorly documented. As a result, its clinical significance remains poorly understood^8,9^.

This study aimed to investigate the timing of the initial radiographic appearance and temporal distribution of PS loosening after lumbar interbody fusion. Given the limited data available on this phenomenon, we sought to clarify when it occurs and how it changes over time. To achieve this, we analyzed the timing and time-dependent variations in PS loosening as observed on CT in patients who underwent single-level posterior lumbar interbody fusion (PLIF) or transforaminal lumbar interbody fusion (TLIF) with PS fixation for degenerative lumbar disease.

## Materials and methods

### Study design and Patients

**Fig. 1:**
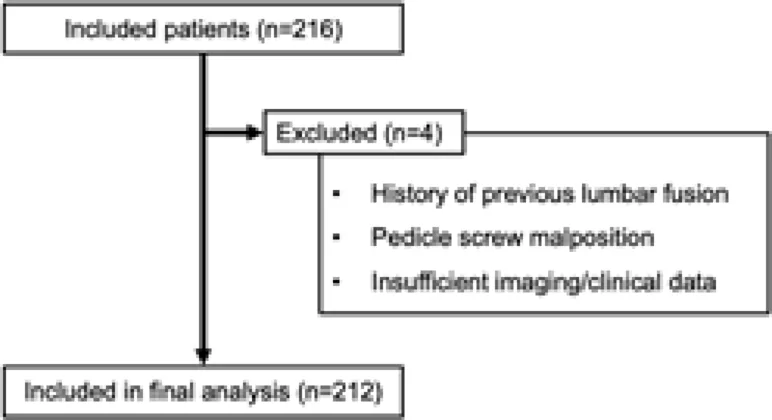
Flow diagram of patient selection.

This study included patients who underwent single-level PLIF or TLIF with PSs and cages at Mie Chuo Medical Center, Mie Prefectural Medical Center, Mie University Hospital, Suzuka Kaisei Hospital, and Yachiyo Hospital between October 2015 and July 2023. Patients were eligible for inclusion if they were 18 years of age or older and had undergone this procedure. Additionally, only those who had undergone preoperative CT examinations and follow-up CT scans with a slice thickness of 0.5–1.0 mm were included. Exclusion criteria were a history of previous lumbar fusion, spinal tumors, spinal infections, vertebral osteolytic lesions, malposition of pedicle screws or cages, or insufficient imaging or clinical data. Of these, four patients were excluded during the study period for the following reasons: history of previous lumbar fusion (*n* = 1), pedicle screw malpositioning (*n* = 1), and insufficient imaging or clinical data (*n* = 2) (Fig. 1).

A total of 212 patients and 552 follow-up imaging studies were analyzed. Multiple follow-up CT scans were performed in a substantial number of cases. Preoperative CT imaging was performed as part of routine preoperative assessment for surgical planning, including evaluation of bony anatomy and instrumentation feasibility, and was not limited to specific grades of spondylolisthesis. Postoperative CT scans were obtained according to institutional practices. In many centers, CT was performed at approximately 1 year postoperatively as part of semi-routine follow-up; however, the timing and frequency of imaging were not standardized and varied among participating institutions. In addition, CT was sometimes performed based on clinical indications such as persistent pain or other postoperative concerns. All CT scans were performed with patients in the supine position. Although the timing of follow-up varied among cases, all patients underwent at least two postoperative CT scans, enabling evaluation of the timing and temporal distribution of screw loosening. The study followed the STROBE guidelines for reporting observational studies.

## Surgical technique

All surgical procedures were performed by spine surgeons certified by the Neurospinal Society of Japan. All screws used were polyaxial, allowing multidirectional movement of the screw heads. After rods and set screws were placed, the screw shafts and heads were securely fixed. During the procedure, the screw heads and rods were also firmly locked into place.

Screw shafts and set screws were composed of titanium alloy, whereas screw heads and rods were made of either titanium alloy or cobalt-chromium. The cage materials consisted of either titanium alloy or polyetheretherketone. Autologous bone graft or artificial bone was packed into the cage and placed around it as well.

## Assessment of PS loosening

**Fig. 2:**
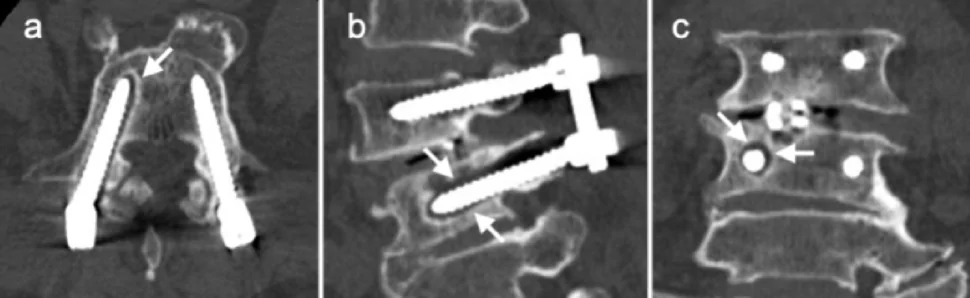
Assessment of pedicle screw (PS) loosening. (**a**) Axial, (**b**) sagittal, and (**c**) coronal CT images. PS loosening was defined as the presence of a radiolucent zone (arrows) measuring ≥ 1 mm in width surrounding the screw, observed in at least one CT plane.

PS loosening was defined as the presence of a radiolucent zone of ≥ 1 mm around the screw on follow-up CT scans (Fig. 2)^10,11^. The CT scans were evaluated by three board-certified spine surgeons in an independent assessment. In cases where no consensus was reached, a decision was made by majority vote.

All scans were evaluated, and cases with a radiolucent zone were identified. Furthermore, to assess the timing of initial appearance and temporal distribution of PS loosening, all CT images of cases with such zones were analyzed, and the imaging time points, as well as the presence or absence of a radiolucent zone, were recorded.

### Statistical analysis

A comparison of patient characteristics between the PS loosening group and the non-PS loosening group was performed using the chi-square test. Continuous variables were compared using an independent t-test. A p-value of < 0.05 was considered statistically significant. All statistical analyses were conducted using EZR (Saitama Medical Center, Jichi Medical University).

## Ethics declarations

This study was approved by the Institutional Review Board of Mie Chuo Medical Center (Approval No. 2023-37). All procedures were conducted in accordance with the Declaration of Helsinki. The requirement for individual informed consent was waived due to the retrospective nature of the study and the use of de-identified clinical data.

## Results

All follow-up CT scans obtained throughout the observation period were evaluated, and PS loosening was diagnosed in 40 cases (18.9%). The median follow-up duration for these 40 cases was 502 days [364.5-731.75]. The characteristics of the PS loosening and non-PS loosening groups are presented in Table 1. A comparison between the two groups revealed that the average age was significantly higher in the PS loosening group. Additionally, a significant difference was noted in the distribution of fixed levels. PS loosening was observed at neither L1-2 nor L2-3 (0%). The highest incidence was at L5-S1 (38.9%), followed by L4-5 (17.2%) and L3-4 (13.3%). No significant differences were observed between the two groups regarding sex, surgical indication, or surgical procedures.

**Table 1:** Characteristics of the PS loosening group and non-PS loosening group.

	PS loosening group^a^	non-PS loosening group^b^	*P* value
	*N* = 40	*N* = 172	
Follow-up period, days	502 (364.5-731.75)	311.5 (164.75-400.25)	< 0.001*
Age, years	70.8 ± 10.8	65.5 ± 11.2	0.007*
Sex (male), n (%)	24 (60.0)	98 (57.0)	0.859
Surgical indication			0.179
Canal stenosis	9 (22.5)	28 (16.3)	
Disc herniation	5 (12.5)	39 (22.7)	
Degenerative spondylolisthesis	20 (50.0)	93 (54.1)	
Spondylolytic spondylolisthesis	6 (15.0)	12 (7.0)	
Fixed level			0.007*
L1-2	0 (0.0)	1 (0.6)	
L2-3	0 (0.0)	14 (8.1)	
L3-4	6 (15.0)	39 (22.7)	
L4-5	20 (50.0)	96 (55.8)	
L5-S1	14 (35.0)	22 (12.8)	
Surgical procedures			0.294
PLIF	21 (52.5)	74 (43.0)	
TLIF	19 (47.5)	98 (57.0)	

Data are presented as mean±standard deviation or number (%), except for follow-up period, which is shown as median (IQR).

*PS* pedicle screw, *PLIF* posterior lumbar interbody fusion, *TLIF* transforaminal lumbar interbody fusion, *IQR* interquartile range.

*P < 0.05 was considered statistically significant.

^a^ Patients with a radiolucent zone ≥ 1 mm around the pedicle screw detected on follow-up CT are considered the PS loosening group.

^b^ Those without evidence of screw loosening are classified as the non-PS loosening group.

**Fig. 3:**
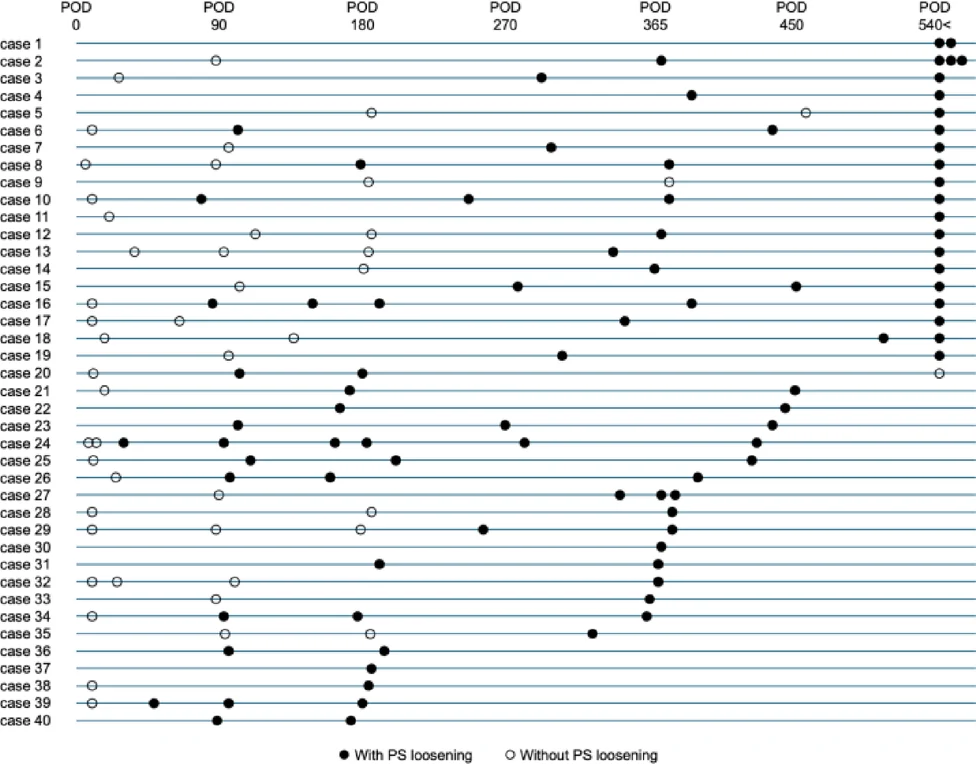
The temporal distribution in imaging findings in cases with pedicle screw (PS) loosening. Follow-up CT scans were plotted according to postoperative days (POD), indicating the presence or absence of PS loosening. Solid circles represent time points with PS loosening, and open circles represent those without.

In cases where PS loosening was identified, the timing of CT scans was plotted to illustrate the initial detection and temporal distribution over time (Fig. 3). Among these cases, approximately half of the patients who underwent CT evaluation by 90 days postoperatively exhibited PS loosening, and by 180 days, it was present in two-thirds of those evaluated by that time. Furthermore, PS loosening continued to appear beyond 180 days postoperatively, with additional cases newly identified up to one year. However, occurrences after one year were rare. None of the patients with PS loosening developed notable symptoms or required revision surgery, and all were managed conservatively.

**Fig. 4:**
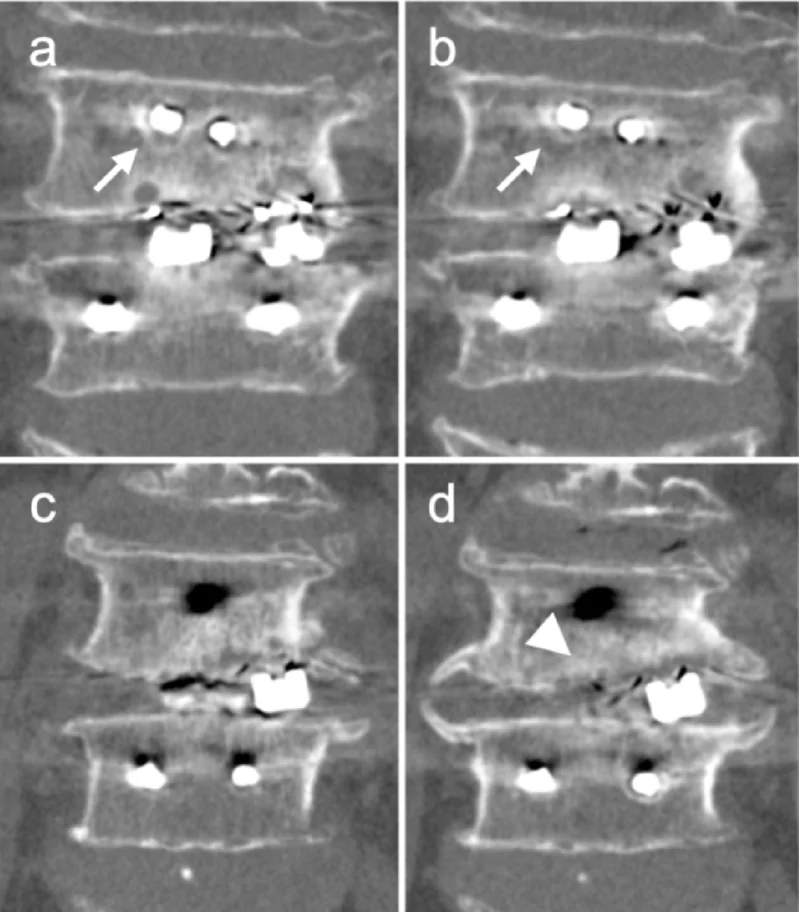
Temporal changes in the radiographic appearance. (**a**, **c**) CT images obtained on postoperative day 177 demonstrating a radiolucent zone around the pedicle screw (arrow). No evidence of bone bridging is observed at this time point. (**b**, **d**) CT images obtained on postoperative day 555 showing a reduction in the size of radiolucent zone (arrow) to below the 1-mm threshold used to define loosening. Evidence of bone bridging (arrowhead) is also observed.

During the follow-up period, a change in the radiographic appearance of the radiolucent zone was documented in only one case. In this case, a radiolucent zone was initially detected on postoperative day 100 and persisted until day 177. However, a CT scan performed on day 555 demonstrated a reduction in the size of the radiolucent zone, falling below the 1-mm threshold used to define loosening. Evidence of bone bridging was also observed (Fig. 4).

## Discussion

PS loosening after spinal fusion surgery is a well-recognized clinical issue. Despite its clinical significance, the timing of its initial radiographic appearance and temporal distribution of PS loosening have not been thoroughly investigated using CT imaging. Therefore, the present study was designed to characterize these aspects using CT. In this study, CT imaging allowed for detailed assessment of screw status, which may have contributed to identifying PS loosening at relatively early postoperative time points. Our findings demonstrated that among cases with PS loosening, approximately half exhibited loosening within 90 days postoperatively, and two-thirds within 180 days. Furthermore, the occurrence of PS loosening after one year postoperatively was rare. These findings suggest that radiographic PS loosening does not necessarily indicate clinical deterioration and should be interpreted in conjunction with the patient’s clinical status.

Several previous studies assessed PS loosening at six months postoperatively. These studies showed that it was present in a certain proportion of cases at that time point; however, detailed information regarding its exact timing is lacking^7^. Similarly, Ninomiya et al. suggested that screw loosening might occur earlier than six months postoperatively in fusion surgery using cortical bone trajectory screws^12^. However, their study did not specifically investigate the occurrence of screw loosening within this period^2,4,7,12^. In this study, PS loosening was already identified in approximately half of the patients evaluated by 90 days postoperatively, suggesting that the loosening process may begin earlier than previously recognized. This observation provides insight into the temporal characteristics of PS loosening and suggests that earlier recognition may be possible. The use of CT in this study likely increased the sensitivity of detection; however, the true stage of loosening at each time point remains uncertain. Spinal fusion is a gradual biological process in which early bone healing is observed around 3 months postoperatively, bony bridging approaches completion by approximately 6 months, and maturation occurs by 12 months. Therefore, in the early postoperative period, fusion is still in progress, and the relationship between mechanical stability and fusion status may not yet be clearly established^6^. On the other hand, previous studies have suggested that PS loosening occurring within six months postoperatively is thought to reflect a decrease in initial fixation strength^7^. Patients with lower initial fixation strength may be at higher risk of progressive loosening, and identifying it at an earlier stage could provide clinical benefits by preventing progressive loosening, reducing the risk of implant failure, and minimizing associated complications. Previous studies have reported that radiolucent zones around pedicle screws may decrease or even disappear over time in association with the progression of bone fusion^7^. Therefore, radiolucent zones do not necessarily indicate persistent loosening, as their appearance may change over time with the progression of bone fusion. This is consistent with our observation that changes in radiolucent zones were not always progressive over time.

Earlier studies have reported that screw loosening predominantly develops within the first six months postoperatively, with late-onset cases considered rare^13,14^. In contrast, in this study, PS loosening was found in approximately two-thirds of cases at 180 days postoperatively, and an additional one-third of cases developed new PS loosening after 180 days and within one year postoperatively. This suggests that PS loosening can occur even after the early postoperative phase, highlighting the need for extended postoperative surveillance beyond the early phase, as delayed cases may otherwise be overlooked. In addition, when radiolucent zones are observed, closer clinical follow-up may be warranted. These findings may reflect changes in fixation; therefore, additional imaging evaluation may be considered if symptoms worsen.

This study has several limitations. First, because this study included multiple institutions and examined surgical cases for various types of lumbar degenerative diseases, the surgical indications were not strictly standardized. In addition, the timing of follow-up CT scans was not standardized across cases. Postoperative CT was performed according to institutional practices, including both semi-routine imaging and imaging based on clinical indications, and the timing and frequency of imaging varied among participating institutions. This variability may have influenced the detection rate and timing of radiolucent zones. At the same time, this variability reflects real-world clinical practice across multiple institutions. Furthermore, some cases lacked long-term imaging follow-up beyond two years. In addition, this study did not evaluate the relationship between radiographic findings and clinical or functional outcomes. Prospective, comparative studies with larger sample sizes are warranted to further clarify these relationships. Consequently, this study cannot completely rule out the possibility that the radiolucent zone may change over extended follow-up periods. Furthermore, since this study utilized CT for evaluation, radiation exposure was relatively high, limiting the feasibility of frequent assessments.

## Conclusions

This study demonstrated that PS loosening occurred in approximately half of the cases within 90 days postoperatively and in two-thirds within 180 days. Although most cases were detected by 90 days, PS loosening remained common throughout the first 180 days. Furthermore, additional cases were newly identified beyond this period, highlighting the importance of extended follow-up. CT imaging facilitated the detection of PS loosening at earlier postoperative time points, which may support improved postoperative surveillance and management. These findings offer new insights into the timing and temporal distribution of PS loosening, which had not been well defined in previous studies. These findings suggest that radiographic PS loosening does not necessarily indicate clinical deterioration and should be interpreted in conjunction with the patient’s clinical status.

## Data Availability

The datasets generated and/or analysed during the current study are available from the corresponding author on reasonable request.
